# Heart rate variability as a strain indicator for psychological stress for emergency physicians during work and alert intervention: a systematic review

**DOI:** 10.1186/s12995-021-00313-3

**Published:** 2021-06-29

**Authors:** Beatrice Thielmann, Robert Pohl, Irina Böckelmann

**Affiliations:** Institute of Occupational Medicine, Faculty of Medicine, Otto-von-Guericke-University, Magdeburg, Leipziger Str. 44, (Building 20), 39120 Magdeburg, Germany

**Keywords:** Heart rate variability, Workload, Mental stress, Emergency physician, Alert, Rescue

## Abstract

**Background:**

The workloads of emergency physicians are severe. The prevalence of burnout among emergency physicians is higher than with other physicians or compared to the general population. The analysis of heart rate variability (HRV) is a valid method for objective monitoring of workload. The aim of this paper is to systematically evaluate the literature on heart rate variability as an objective indicator for mental stress of emergency physicians.

**Methods:**

A systematic literature review examining heart rate variability of emergency physicians in accordance with the Preferred Reporting Items for Systematic Reviews and Meta-Analysis (PRISMA) statement for reporting systematic reviews was performed. PubMed, Ovid, Cochrane Libary, Scopus, and Web of Science electronic databases were used. The methodological quality was evaluated by using a modified STARD for HRV.

**Results:**

Two studies matched the inclusion criteria by using HRV between alert intervention and two other studies were considered that used HRV in other question areas. It showed an adaptation of HRV under stress. The studies were not comparable.

**Conclusions:**

There is a need for occupational health studies that examine strains and stress of emergency physicians. The well-established parasympathetic mediated HRV parameters seem to be suitable parameters to objectify the stress.

## Abstract (de)

**Hintergrund:**

Die Arbeitsbelastungen von Notärzten sind hoch. Die Prävalenz von Burnout ist bei Notärzten höher als bei anderen Ärzten oder im Vergleich zur Allgemeinbevölkerung. Die Analyse der Herzfrequenzvariabilität (HRV) ist eine valide Methode zur objektiven Überwachung der Arbeitsbelastung. Ziel dieser Arbeit ist eine systematische Auswertung der Literatur zur Herzfrequenzvariabilität als objektiver Indikator für die psychische Belastung von Notärzten.

**Methoden:**

Es wurde eine systematische Literaturrecherche zur Herzfrequenzvariabilität von Notärzten in Übereinstimmung mit dem Preferred Reporting Items for Systematic Reviews and Meta-Analysis (PRISMA) Statement zur Erstellung systematischer Reviews durchgeführt. Es wurden die elektronischen Datenbanken PubMed, Ovid, Cochrane Libary, Scopus und Web of Science verwendet. Die methodische Qualität wurde mit Hilfe eines modifizierten STARD für HRV bewertet.

**Ergebnisse:**

Zwei Studien erfüllten die Einschlusskriterien, indem sie die HRV zwischen Alarminterventionen verwendeten und zwei weitere Studien wurden berücksichtigt, die die HRV in anderen Fragebereichen verwendeten. Es zeigte sich eine Anpassung der HRV unter Stress. Die Studien waren nicht vergleichbar.

**Schlussfolgerungen:**

Es besteht ein Bedarf an arbeitsmedizinischen Studien, die Belastungen und Stress von Notärzten untersuchen. Die gut etablierten parasympathisch vermittelten HRV-Parameter scheinen geeignete Parameter zu sein, um die Belastung zu objektivieren.

## Background

Emergency physicians are exposed to many strains in their workday. Examples include time pressure, necessity for rapid decisions, violence by patients or relatives, death of the patient, traumatic events and shift work [1]. The current report of the performance level in the public ambulance system shows an increase in emergency operations. In the process, emergency physician services increased from 1.45 million in 1994/1995 to 3.0 million in 2016/2017 [2]. A retrospective analysis from Austria showed that a strict indication for emergency physician intervention was only necessary in 17.1% of the alerts [3]. Those measures would be intensive care, intubation, ventilation or catecholamine therapy. Consequently, the authors reported a high number of calls with emergency physician, who were not needed during the rescue operation. This can further increase the level of frustration as another workload for emergency physicians.

Definitions of workload are not standardized. Workload can be defined as the balance between work related task and a person’s response to that task [4]. The concept “Stress and Strain” from Rohmert and Rutenfranz [5] was defined with regard to stressors/workloads associated with physiological strain/stress reaction. The different types of stressors lead to the different strain of each individual. Therefore, under the term “strain” is meant the response stress reactions of the organism to stressors [5]. Thus, stressors are typical work-related factors that lead to a stress reaction as a short-term consequence of stress. This stress reaction can be positive and negative.

If stress continues without adequate compensation, adverse health effects can occur. These long-term negative effects include, for example, the increased incidence of cardiovascular disease, depression or burnout [6–8]. Therefore, burnout is always a long-term negative consequence of stress. The World Health Organization defines burnout according to ICD-11 as “…a syndrome conceptualized as resulting from chronic workplace stress that has not been successfully managed…” [9]. Work absenteeism due burnout is increasing [10]. There is great interest in occupational medicine among physicians to counteract mental diseases caused by work stress. Mental diseases, such as burnout doesn’t just affect the health of the physician, it also affects the safety of the patient. Typical job stressors were more highly correlated with strain than with burnout. Job importance was more highly correlated with burnout than with strain [11], which is prominent among physicians [12]. The Medscape National Physician Burnout & Suicide Report 2020 reported, that 43% of the emergency physicians (42% of all physicians) had a burnout that year [13]. Thus, the great importance of an objective measurement of subjective stress follows. It’s important for research, occupational medicine and clinical practice [14].

The analysis of heart rate variability (HRV) is a possible method for objective monitoring of workload [15]. HRV is often used for stress identification in surgeons [16, 17]. The HRV is defined as variations in time between consecutive heart beats and it’s a very sensitive indicator for dysregulation of the autonomic nervous system (ANS) [18, 19]. In order to make statements about the degree of stress and the quality of regulation of the cardiovascular system possible, the analysis of HRV is considered an established non-invasive recording method in occupational medicine and occupational sciences due to increasingly smaller measuring instruments and lower costs [20]. HRV analyses can also be used to extend questions from the fields of health promotion, workload management and stress management [21, 22].

**Fig. 1:**
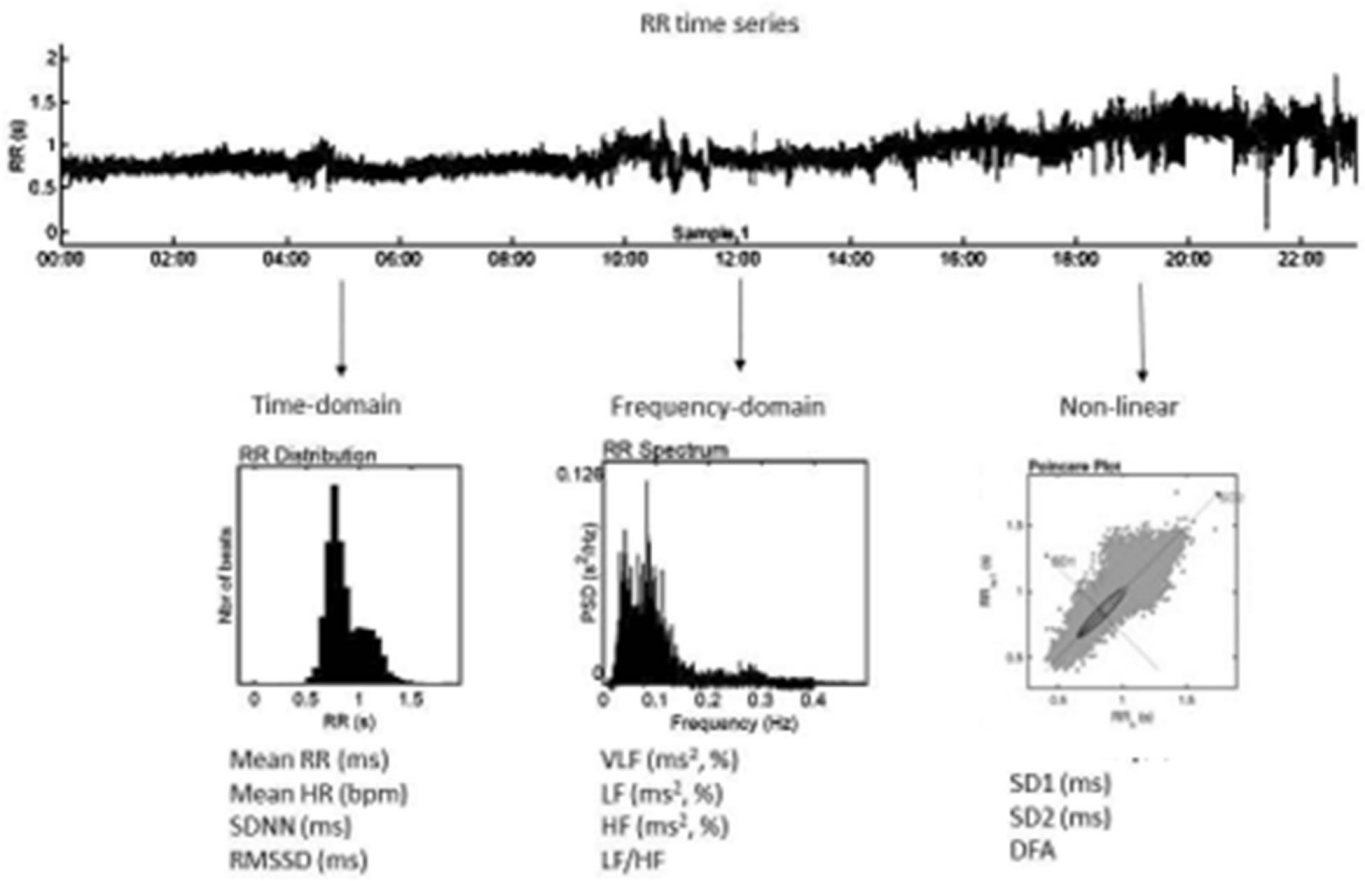
Different domains of Heart Rate Variability

HRV is based to a significant degree of the tone of the vagus nerve, which excites the atria of the heart and modulates the self-sustained sinus rhythm of the sinus or Keith flack node. Due to the resulting interrelation between the sympathetic and parasympathetic nervous systems, HRV analyses can be used to estimate different demands in a more differentiated manner [18]. Especially in body rest and recovery phases, parasympathetic activity predominates and in chronic stress state, sympathetic activity dominates [18]. HRV analysis can be differentiated based on time, frequency and nonlinear domains. Time domain values measure how much HRV was observed during the monitoring period. Frequency domain values calculate absolute or relative signal power within the ultra low frequency (ULF), very low frequency (VLF), low frequency (LF) and high frequency (HF) bands. Nonlinear parameters measure the unpredictability and complexity of a series of interbeat intervals [23]. Figure 1 shows the three domains of HRV measurements. The ANS has an important role in stress regulation, so that chronic (work-related) stress has been associated with reduced HRV and reduced parasympathetic modulation [24]. The HRV parameters, for example the root mean square of successive differences (RMSSD), percentage of successive NN intervals that differ by more than 50 ms (pNN50), high frequency spectrum (HF, HF%, HFnu) and standard deviation of point plot to the transverse diameter (SD1) are established markers of vagal function [18]. In vagal tone (activity of the vagus nerve), the parasympathetic nervous system predominates and this is proposed as a novel index of stress and stress vulnerability [25]. In contrast, other parameters (e.g., LF, LF/HF) are without clear assignment and can be influence from the sympathetic and parasympathetic nervous systems. Furthermore, it is also necessary to consider which recording time is necessary (e.g., 24-h, short-term (5 min), and ultrashort-time (< 5 min) for according parameters and which parameters are relevant for the question to be determined [26]. An overview of HRV metric is given by Shaffer and Grinsberg 2017, Sammito and Böckelmann 2015 or the current guidelines [18–20, 23]. Finally, it should be noted that HRV is age-related [27–29], so that reference values for the age should be used [30]. The first reference values have been available for only a few years.

The aim of this project is to systematically evaluate the literature on heart rate variability as an objective indicator for mental stress in emergency physicians during their shift work. The focus was on the objective measurement of stress during an alarm operation in comparison to another working day without alarm operation. The alarm (alert) operation is considered as special stress with corresponding strain, because it leads to the directly interruption of the activity after the alarm with directly mission trip to the place of the emergency call. We hypothesized that in the working phase there is an increased reduction in vagal tone. It is necessary to keep certain time limits for rescue and to save the life of the emergency patient. Shift work means that several people are assigned to the same workplace to perform their work at different times within a period of time. In the context of emergency medicine, this would be work in emergency rescue, which although can also be 24 h (including night work) and is different from the normal working day.

## Methods

We performed a systematic literature review that examined heart rate variability in emergency physicians in accordance with the Preferred Reporting Items for Systematic Reviews and Meta-Analysis (PRISMA) statement for reporting systematic reviews [31].

The electronic databases PubMed, Ovid, Cochrane Libary, Scopus and Web of Science were used (deadline: September 20, 2020). As Search terms were defined “emergency physician” OR “emergency doctor” OR “doctor on call” OR “helicopter doctor” OR “helicopter physician” AND “heart rate variability” OR “HRV” OR “cardiac autonomic control” OR “autonomic function” OR “parasympathetic activity” OR “parasympathetic nervous system” OR “cardiac vagal tone” OR “autonomic cardiac modulation” OR “vagus nerve” OR “vagal tone” OR “vagal activity” OR “coefficient of variation”. Only articles since January 1st, 2005 were included.

Inclusion criteria were physicians with certification in emergency medicine, more than 10 participants (in each group), measurement of HRV before (after) and during working hours or alarm intervention, recording of heart rate through Holter ECG or chest belt, clear statement of the data treatment for abnormal or ectopic beats and full-text in English or German language. A non-transmitting memory belt (chest belt) is suitable for recording RR intervals and the heart rate, but not for HRV measurement under high exercise conditions. A high degree of conformity with the Holter ECG system was showed [18, 32]. The recordings during the work or alarm operation are to represent the strain of the emergency physicians during the alarm operation in comparison to an ordinary working day. If possible, a comparison should be made in leisure time.

Exclusion criteria were diagnosis of mental or neurological diseases, endocrinological diseases (diabetes, thyroid gland disease), cardiac diseases, hypertension, other HRV-analysis-related diseases, review articles, guidelines, single-case-studies, theses, dissertations, scientific conference abstracts and HRV assessment with pulse rate automatic or photoplethysmography.

In contrast to an Holter ECG to measure RR intervals, the measurement of the pulse rate is affected by further factors (e. g. vascular stiffness) [33]. Pulse oximeters are suitable for measuring RR intervals (named as pulse rate variability (PRV)) to a very limited degree only [34]. Particularly the high frequency band is subject to overestimates in PRV compared with HRV [35]. The national guideline on HRV do not suggest this method of measurement [18].

Following the HRV guidelines, we used only heart rate variability and not pulse waves due photoplethysmography [18, 19]. The last method identifies only pulse waves per minute and is recorded periphere (e.g., at the wrist). A difference between the two methods is possible because, for example, ineffective heart contractions occur in certain forms of cardiac arrhythmia, resulting in a pulse beat that cannot be measured (pulse deficit). Thus, the two methods are not comparable.

**Fig. 2:**
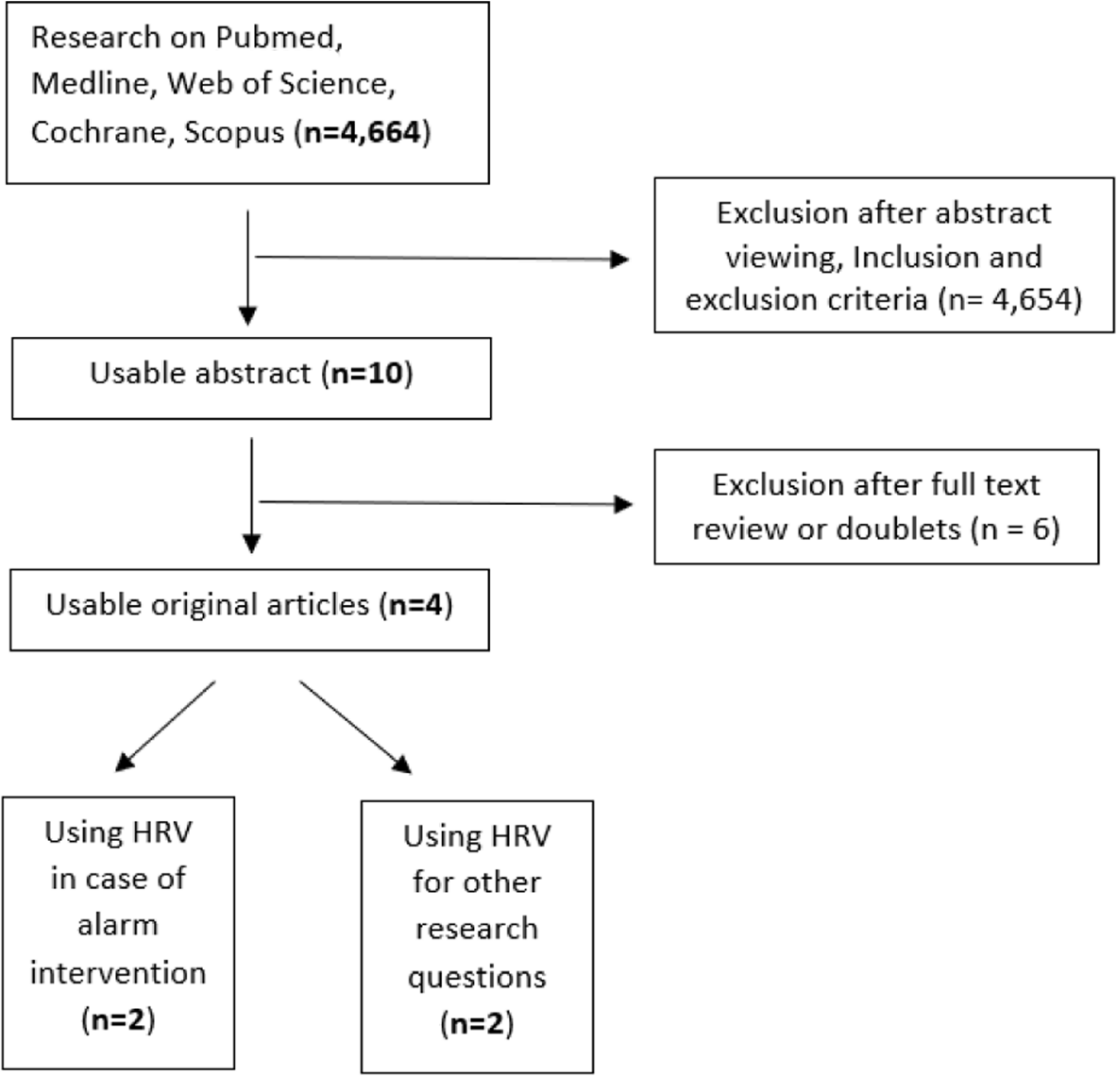
Procedure in the context of the systematic literature search

In addition, a hand search was performed by checking the reference lists of the included studies (no result). An overview of the procedure shows Fig. 2. The complete study protocol is available at Prospero (https://www.crd.york.ac.uk/PROSPERO/display_record.php?RecordID=210274). Because of the small number of cases, we also evaluated HRV analyses for this review, which were not only based on rescue operations, but also on other research questions.

The retrieved articles were transferred to the Citavi 6 reference manager (Swiss Academic Software, Wädenswil, Switzerland). Duplicates were removed. Two authors (B.T. and R.P.) independently screened titles and abstracts according to the inclusion and exclusion criteria. Thereafter, the full-text of each relevant article was obtained. The same two authors independently screened the full-text of these articles. If no full-text was available, the authors were contacted. The references of the eligible articles were screened to retrieve further articles. Disagreements were resolved through discussion with a third reviewer (I.B.).

The methodological quality of the included studies was evaluated using the Standard for Reporting Diagnostic Accuracy Studies (STARD) guidelines [36, 37]. It follows the recommendations of [26, 38]. All studies were also evaluated independently by two authors (B.T. and R.P.) using a modified STARD for HRV by [39]. It included 25 items, which are shown in Table 1. Accordingly, a maximum of 25 points could be achieved. We have also slightly modified two assessment tools according to [40]. However, the maximum score did not change. Disagreement was solved by discussion.

**Table 1:** Standard for reporting diagnostic accuracy studies guidelines for heart rate variability research (STARD_HRV_) by (Bossuyt et al. 2003 [36]; Cohen et al. 2016 [37]; Quintana et al. 2016 [38]; Dobbs et al. 2019 [39]; Laborde et al. 2017 [26])

	Evaluation point
1	Identification as a study of validation
2	Structured summary of study objective, design, methods, results and conclusions
3	Scientific and practical background, including the intended use of the index device/software
4	Study objectives and hypotheses described
5	Study uses within-subject design
6	Intended sample size and how it was determined (e.g. G*Power 3)
7	Eligibility criteria including specific restrictions (medical use, gender, age, activity level or BMI)
8	Pre-testing guidelines reported (e.g., limitations to caffeine, alcohol, physical activity etc.)
9	Setup of reference standard and index device described in sufficient detail to allow replication (e.g. hardware/software such as brand, electrode configuration, etc.)
10	Description of environmental conditions (e.g. temperature, humidity, lights on or off, time of day) and posture
11	A stabilization period prior to sampling was described
12	The raw sampling rate and length of collection are described
13	Acknowledgment of breathing (e.g. controlled or not controlled)
14	Description of how estimates or comparison measures were calculated (e.g. ES, LOA, Pearson’s r or ICC)
15	Reasons for missing data, along with percentage missing (e.g., equipment, persistent ectopy) and how it was handled
16	Interbeat artifact identification method (e.g. algorithm, manual inspection)
17	Artifact cleaning methods and percentage of beats corrected
18	Description of metrics used and software/script for HRV calculation (log transformation etc.)
19	Specification of frequency bands used and how they were calculated (e.g. Fast Fourier Transform or Autoregressive modelling)
20	Baseline demographics of participants
21	Mean ± SD along with at least one estimate of precision (e.g. LOA, Pearson’s r or ICC)
22	Study limitations (e.g., sources of potential bias, confounding variables, statistical uncertainty and generalisability)
23	Implications for practice, including the intended use
24	Where the full study protocol can be accessed if not fully described
25	Sources of funding and other support; role of funders

From four studies, the change of all used HRV parameters were collected. Increases were marked with an upward arrow, decreases with a downward arrow and no change with an arrow pointing to the left and right. Significant changes were marked with an asterisk.

## Results

The initial search resulted in 4664 records. After removing duplicates and excluding based on title and abstract only 10 full-texts were assessed for eligibility. Finally, 2 studies matched the inclusion criteria by using HRV between alert intervention. One of these studies examined helicopters emergency physicians [41]. Two other studies were considered that used HRV in other question areas (one burnout and one shift work in emergency physicians). An overview of the four included studies shows the Table 1.

Two of the included studies using HRV between alert intervention comprised a total of 33 subjects [41, 42]. The other 2 studies, using HRV in other question areas, included 63 participants [43, 44]. One between-subject-design was used, which examined between groups of burnout with and without alarm signs [44]. Only one study excluded emergency physicians with current and endocrine diseases, pregnancy or chronotropic drugs [43]. One study provided information about smokers [41], two studies gave details of body mass index [41, 43] and another study made notes about medication intake [41].

The studies using HRV between alarm intervention utilized chest belts, but one recorded over 24 h [42] and one during the working time without further information about the shift duration [41]. The both studies using HRV in other question areas also tracked HRV over 24 h [43] or during the daily duties, also without information about the duration [44]. No further information on the ECG lead was provided. Two studies evaluated cardiac autonomic function by time-domain and frequency-domain [41, 43], a third study used in addition non-linear HRV parameters [42]. More novel parameters were used by [44]. None of the studies used short-term. None of the studies used short-term measurements of heart rate variability as baseline [41–44]. Although the 24-h and short-term HRV measurements using the same methods, they are not substitutes for each other, and their physiological importance could be fundamentally different. Therefore it would be preferable [23].

### Outcome heart rate variability

Table 2 presents the outcome of all HRV measures. All studies reported significant positive adaptations in HRV during the monitoring phase. Equally, all studies used RMSSD as a marker of vagal function. RMSSD decreased during emergency operations, compared to the control day [41] or pre-alert period [42]. RMSSD increased again after primary care time but did not reach baseline (no significance) [42]. The same was seen for the SDNN parameter [41, 42]. In comparison between clinic day and day as an emergency physician, SDNN and RMSSD increased and LF/HF decreased here [41]. Sympathetic activation in alarm operation shows the increase of LF and LFnu as well as the decrease of HF and HFnu [42]. The nonlinear parameters SD1 (parasympathetic) and SD2 (symphathetic and parasympathetic) decreased [42]. Sympathetic activation was performed during a 24 or 14 h shift compared with a clerical day. HRV was more prominent during a 14 h shift than during a 24 h shift [43].

**Table 2:** Outcome and measurement of HRV, characteristics of subjects and results of STARD HRV

Author, year	method	Outcome and measurement of HRV	Characteristics and risk factors of subjects	STARD HRV
using HRV between alert intervention				
Petrowski et al., 2019 [41]	ECG at shift, chest belt, Within	*Clinical-Day and Air-Day:* HR↑, SDNN↑, RMSSD↔, LF/HF↓*Control-Day and Air-Day: HR↔: SDNN↓, RMSSD↓, LF/HF↔	Age 44.95 ± 4.8 years, *n* = 20, females = 3, males = 17, BMI Ø 26.39 kg/m^2^, smokers = 3	16.5
Schneider et al., 2017 [42]	24-h ECG, chest belt, Within	*before and primary care time:* SDNN↓**, RMSSD↓*, pNN50↓*. LF%↑, HF%↓*, LF/HF↑*, LFnu↑**, HFnu↓**, LF↑, HF↓*. SD1↓*, SD2↓**, PeEn↑**, ApEN↑**, SampEn↑**, ShanEn↓**, D2↑*	Age mean 38.4 years, *n* = 13, females = 2, males = 11, recordings *n* = 23,	15
Using HRV in other question areas				
Dutheil et al., 2012 [43]	24-h-ECG, within	*24 h or 14 h shift and clerical day without patient contact (control day):* 24 h: RMSSD↓*, LogLF/HF↑*. 14 h: RMSSD↓, LogLF/HF↑*. *24 h and 14 h:* RMSSD 14 > 24 h*, LogLF/HF 24 > 14	Age 39.1 ± 6.9 years, *n* = 19, females = 12, males = 7, BMI Ø 22.8 kg/m^2^,	8.5
Kotov et al., 2012 [44]	ECG at work, between	Comparison Burnout syndrome (BS): no signs 0 and alarm stage I in groups of task-oriented or emotion-oriented behavorial coping strategy. *Task-oriented:* SDNN↓*, RMSSD↓*, pNN50↓, VLF↑*, LF↓*, HF↓*, LF/HF↑, SI↑, CI↑. *Emotion-oriented:* SDNN↑, RMSSD↑, pNN50↑, VLF↓*, LF↓*, HF↓*, VLF↑ *, LF/HF↔, SI↑, CI↑	*n* = 19 (females = 12, males = 7) task-oriented: BS 0 n = 10, BS 1 *n* = 10, emotion-oriented: BS 0 *n* = 15, BS 1 *n* = 9, BS 0 (females = 14, males = 11) BS 1 (females = 11, males = 8)	6.5

within = within-subject-design, between = between-subject-design. *BMI* body mass index. Significant *p*-values are marked with asterisks (* for *p* < 0.05 and ** *p* < 0.001)

HRV parameter: Time domain, *SDNN* standard deviation of all normal-to-normal R-R intervals, *RMSSD* root mean square of successive differences of R-R intervals, *NN50* the number of pairs of successive normal-to-normal R-R intervals that differ by more than 50 ms, *pNN50* percentage of successive NN intervals that differ by more than 50 ms. Frequency domain: *VLF* Very low frequency power, 0.003–0.04 hz, *LFpow* low frequency power, 0.04–0.15 hz, *LFnu* low frequency normalized units, *HF pow* high frequency power, 0.15–0.4 Hz, *HFnu* high frequency normalized units, LF/HF-ratio. Non-linear domain: *SD1 and SD2* standard deviations of the Poincare plot, *PeEn* permutation entropy, *ApEn* approximate entropy, *SampEn* sample entropy, *ShanEn* Shannon entropy of diagonal line lengths’ probability distribution, *D2* correlation dimension. Others: *SI* strain index, *CI* centrilization index

Parasympathetic-associated parameters (RMSSD, pNN50, VLF, HF) decreased in task-oriented emergency physicians with alarm signs of burnout, whereas they increased in emotion-oriented ones [44]. The trend of HRV parameters looks adaptive to the stress situation of emergency physician during alert intervention or working day [41–44]. Satisfactory adaptation to stress is known to involve moderate activation of the control systems to adapt to the modified environment. One study showed a higher RMSSD in the group with burnout alarm stage I and emotion-oriented behavioral coping strategies [44].

### Quality assessment

The study quality of HRV methodology was evaluated with STARD_HRV_ [39]. The score for both studies using HRV between alert intervention was 15 [42] and 16.5 [41]. Those studies using HRV in other question areas achieved noticeably lower points (6.5 [44] and 8.5 [43]).

Faults were found in the case of elevation point 6, 11, 13, 15 and 17 in all studies. Point 8 and 16 were only listed in isolated cases. Monitoring during the work (alarm operation or normal work) could lead to movement artifacts, which limits the assessment. Two studies did not report this [43, 44]. One study claimed to clean up artifacts visually [42]. Another study only stated that artifacts were cleaned up [41]. None of the studies reported the percentages of adjusted material [41–44].

## Conclusions

The main aim of this systematic review was to summarize the existing literature on the heart rate variability in emergency physicians during their work or alert intervention. Especially under the COVID-19 pandemic, emergency physicians and the other rescue service employees are exposed to even more psychological stress than before. Two studies were found that examined HRV during alarm operation, two other studies using HRV in other question areas. The selected HRV parameters are able to provide information about the measured strain. It should be noted that there are different study protocols and different recording times, so that these values are only comparable to a limited degree. Lack of methodological quality and quality of study reports were identified. The subject numbers are very low, so generalization is not necessary. However, it can be said that the predominantly parasympathetic mediated parameters adapt to the workload with a decrease. This is mainly during an emergency intervention compared to before or after the alert or compared to a control day with no patient contact. The same applies to shift work of different lengths compared to an office day. No statements are possible regarding the use of non-linear parameters. They adapt to a workload, but different parameters were used in two studies.

This systematic review shows that there is a high need and a great potential for occupational health studies among the professional group of emergency physicians. Emergency physicians have higher burnout levels (60%) compared with physicians in general (38%) [45]. Despite the high burnout rates among emergency physicians, not much work has been done in this area so far, as the review demonstrates. The factors causing burnout in emergency physicians should be identified and adequate interventions developed to reduce burnout. HRV is a suitable method to objectify subjective stress. The use of a chest belt increases the wearing comfort and seems to be practical especially in emergency rescue operations. HRV parameters without clear association to the ANS or recent parameters should be used in addition to be able to provide better statements in the future. The use of novel physiological monitoring techniques such as transdermal optical imaging (TOI) to assess basal stress is also feasible in the field. This method comfortably and contact-free measures changes in facial blood flow from a distance using a conventional digital video camera [46]. Further validation studies (comparing HRV versus TOI) should be performed for this purpose.

HRV is also used to study stress levels of surgeons during operations. The review by The et al. shows that there are significantly more studies on this topic (*n* = 17) [17]. For example, reviews showed that stressed surgeons (e.g., intraoperatively) offered reduced HRV [16, 17]. The same was observed for ENT physicians performing surgery [47]. High workload and low work control in non-operating resident physicians were also associated with of decreased heart rate variability [48].

In summary, there is a need for occupational health studies that examine strains and stress of emergency physicians. HRV is a valid method for the visualization of stress. Currently, the well-established parasympathetic mediated HRV parameters seem to be suitable parameters to mediate this. HRV parameters without clear association to the ANS or recent parameters should be used in addition. In this context, it also seems useful to have a standardized study protocol (e.g., measurements before, during, and after an alarm intervention and comparison to a normal workday).

## Data Availability

The data can be accessed via the corresponding author. They are archived at the University of Magdeburg.

## Abbreviations

ANS: Autonomic nervous system
ApEn: Approximate entropy
CI: Centrilization index
HF: High frequency spectrum
HR: Heart rate
HRV: Heart rate variability
LF: Low frequency spectrum
LF/HF: Relationship between LF and HF power
pNN50: Percentage of successive NN intervals that differ by more than 50 ms
RMSSD: Root mean square of successive differences
SampEn: Sample entropy
SD1: Standard deviation of point plot to the transverse diameter
SD2: Standard deviation of the distances of the points from the major axis
SDNN: Standard deviation of NN intervals within the measurement area
ShanEn: Shannon entropy of diagonal line lengths’ probability distribution
SI: Strain (stress) index
ULF: Ultra low frequency spectrum
VLF: Very low frequency spectrum
